# Older individuals’ views on their personal screening results for complex health problems: a qualitative study

**DOI:** 10.1186/s12875-020-01280-0

**Published:** 2020-10-19

**Authors:** Sophie C. E. van Blijswijk, Lisa S. van Tol, Jeanet W. Blom, Wendy P. J. den Elzen, Jacobijn Gussekloo

**Affiliations:** 1 Department of Public Health and Primary Care, Leiden University Medical Center, Leiden, The Netherlands; 2 Department of Clinical Chemistry and Laboratory Medicine, Leiden University Medical Center, Leiden, The Netherlands; 3 Department of Internal Medicine, section Gerontology and Geriatrics, Leiden University Medical Center, Leiden, The Netherlands

**Keywords:** Community-dwelling older persons, Screening questionnaire, Self-management, Personal goals, Older individuals’ views

## Abstract

**Background:**

Providing older persons with information about their health status may increase their involvement in their own health and enhance self-management. However, we need a better understanding of how older persons view their personal results after completing a screening questionnaire on complex health, of their (lack of) motivation and their subsequent action.

**Methods:**

In this qualitative study community-dwelling older persons (≥80 years, *n* = 13) who completed a screening questionnaire on complex health problems were interviewed regarding their perception of the results, the actions they considered taking and their personal motivations. Data were analysed thematically (qualitative content analyses).

**Results:**

Participants expressed interest in feedback, as an objective questionnaire might substantiate their own views regarding their personal health. They were mostly unsurprised by the results and/or had already taken precautions and were therefore not inclined to undertake additional action. They admitted difficulty with and appreciated advice from a professional regarding preparation of an action plan. Unexpected negative results would lead them to discuss matters with family and/or their general practitioner, provided they had a good relationship with their GP.

**Conclusion:**

Older people were interested in direct feedback regarding their screening questionnaire results and in subsequent advice on possible additional measures. General practices could consider inviting older persons to complete a screening questionnaire and discuss activities and personal goals. This information could serve to better shape future interventions aimed at increasing self-management amongst older persons.

## Background

A patient-centred approach in which older persons are more actively involved in their own health could help increase self-management and might have a beneficial impact on health [1, 2]. A patient-centred approach also accords with current trends towards shared decision making and personalised healthcare. Indeed, many older persons wish to be informed about their own health, although there is wide individual variation [3]. Results from earlier studies also indicate that they are less inclined than younger people to take decisions concerning their own healthcare [2, 4, 5]. It has been suggested that monitoring one’s own health can stimulate patient involvement in medical decisions [6, 7]. Furthermore, providing older individuals with information about their health status could help motivate them to manage their own health [8, 9]. Despite these promising results, there is currently little consensus on the health benefits of interventions that promote the active involvement of older persons in their own healthcare [2, 10].

It is important to explore the opinions of older persons regarding their involvement in their own healthcare. This can be achieved by providing them with information on their health status. The aim of this study was to contribute to knowledge concerning the active participation of older persons in their own healthcare and to enhance self-management. This knowledge can then be used to shape interventions that increase the involvement and self-management of older persons. In this study we investigated the views of older persons on feedback following completion of a health screening questionnaire (question 1). We also asked whether they felt the need to take action as a consequence and what motivated or limited subsequent action (question 2). Finally, we reviewed actions they considered (question 3).

## Methods

### Study design and population

This qualitative study is embedded within the follow-up of the ISCOPE trial. Detailed information on that trial has been published elsewhere [11]. In short, this cluster randomised trial investigated the cost-effectiveness of proactive care for community-dwelling older persons in general practice. During the inclusion period of the ISCOPE trial (baseline September 2009–September 2010), community-dwelling older persons (≥75 years) received a postal ISCOPE screening questionnaire (Additional file 1) on complex problems, in addition to several additional health questionnaires. A sample of the participants received home visits to obtain additional information. During follow-up (one-year and six-years later) participants completed the ISCOPE screening questionnaire again and home visits were repeated. Participants were asked if they wished to participate in additional studies aimed at improving primary care for community-dwelling older persons, of which this interview study is an example. During the study (2017) all participants were aged 80 years and over.

Of the participants eligible for additional studies, we first contacted those who lived in the direct surroundings of the research facility (in alphabetical order). We used purposeful sampling to capture a broad range of opinions and therefore selected those participants who had experienced an increase in complex problems over the previous 6 years together with those who had not. We also selected both male and female participants. Eligible interviewees were contacted by telephone (March – May 2017) and those who agreed to participate were sent an appointment confirmation and were interviewed at home 2 to 3 weeks later.

All participants provided written informed consent for the ISCOPE follow-up study. Additionally, informed consent for the interview was recorded. The Medical Ethical Committee of Leiden University Medical Center approved the ISCOPE follow-up study, of which the current qualitative study is a part (P15.256).

### Interviews

An interview guide (Additional file 2) was developed prior to the first interview. The semi-structured interviews were performed by either a master’s student in health sciences and vitality & ageing (LST, female) or a GP trainee/PhD student (SCEB, female) in May 2017. Three interviews were conducted jointly to establish whether changes were needed in the interview guide and to learn from each other.

During each interview the results of the most recent and the previous ISCOPE screening questionnaires were supplied to the participant to allow reflection on the results. The ISCOPE screening questionnaire is designed to facilitate the early identification of community-dwelling older persons with complex health problems [11]. The questionnaire covers four domains of health (somatic, functional, psychological and social). Participants were considered to have complex problems when problems were present in three or four health domains. We have previously shown that complex health problems are associated with poorer health [11, 12].

During the interviews we explored possibilities of involving community-dwelling older persons in their own healthcare, and of helping them identify possible solutions to their problems by regularly updating them on their personal health status. Participants were asked their opinions on the screening questionnaire in general and their personal results in particular, and what actions they had considered taking to improve or stabilize their health following these results. Finally, we discussed their reasons to take or not to take action.

All interviews were audio-recorded and notes were made. After each week the content of the interviews was discussed and iterative changes to the interview guide were made if needed. Two to four interviews were performed each week. Data saturation was discussed weekly and no additional interviews were planned after data saturation was reached.

### Data analysis

Participant characteristics including age, sex, marital status, living situation and health domains with problems were obtained from participants via questionnaires at the time of the interview.

Audio records from the interviews were transcribed verbatim. All interviews and corresponding notes were compared and discussed by two researchers (LST and SCEB). Data were analysed thematically (qualitative content analyses) and a list of codes was inductively developed. Three interviews were coded independently by two researchers (LST and SCEB) and a list of codes and quotes per research question was developed based on this. This served as framework for further analysis. The other interviews were further analysed per research question to add data to the previously developed framework of codes and quotes. Quotes were selected (independently by both researchers) and compared with quotes on the same research question from different interviews (by the two researches together). The findings were then discussed with all authors. Data were processed and analysed with Speech Exec pro transcrib and Atlas.ti 7.5.

## Results

### Participant characteristics

**Fig. 1:**
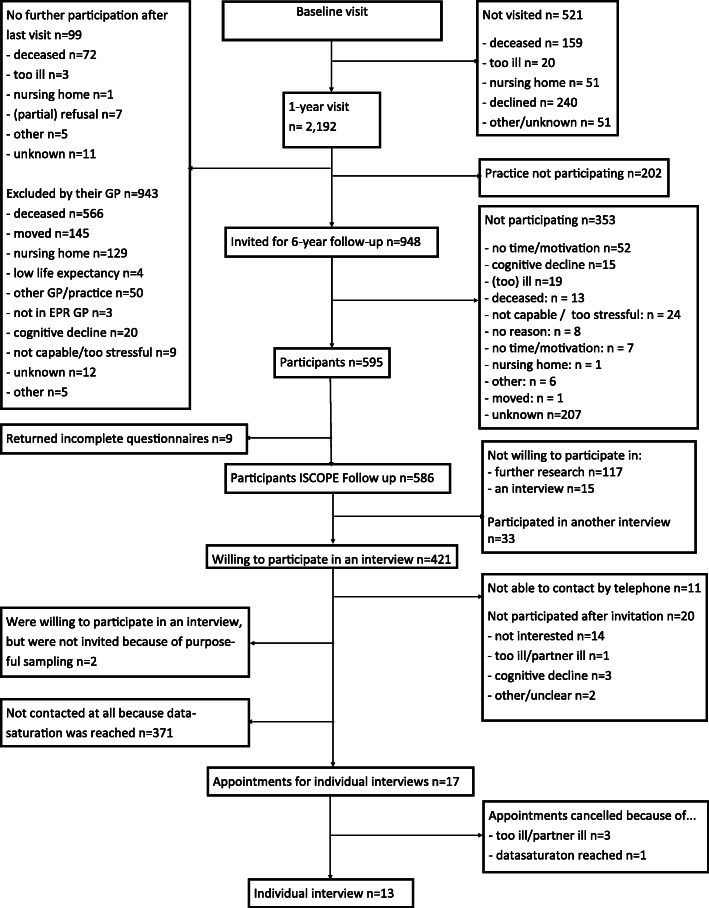
Flowchart of the study, nested in the ISCOPE study

Of the 586 older persons who participated in the follow-up of the ISCOPE study, 421 agreed to participate in an additional study. In total, 50 older persons were contacted by telephone and 19 agreed to an interview. Two did not meet the criteria for purposeful selection for the last interviews (i.e. three of four domains with problems on the ISCOPE questionnaire). Three interviews were cancelled by the participants because of illness. After 13 interviews data saturation was reached (Fig. 1). On average the interviews took 48 min (range 35–68 min).

Three of the thirteen participants were male and the median age was 87.4 years (IQR 84.6–88.0). Eleven participants were widowed. All thirteen participants lived independently, of whom two with their spouse and one with an adult child. Based on the ISCOPE screening questionnaire, seven participants had complex health problems. Two participants had problems on the functional domain and at least eight participants had problems related to the psychological, somatic or social domain (Table 1).

**Table 1:** Patient characteristics at interview (*n* = 13)

Variable	N
Age in years (median)	87.4 (range 83–91)
Female	10
Marital status	
Widowed	11
Married	2
Living situation	
Independent, alone	10
Independent, with child/partner	3
Complex problems according to ISCOPE	7
No complex problems	6
Problems on a health domain	
Functional	2
Somatic	8
Psychological	8
Social	9

### Results of the interviews

Interview results are presented per research question. We first describe the participant’s opinions on the ISCOPE screening questionnaire and then describe their views on receiving the results of the screening questionnaire (question 1). Subsequently, we describe whether the participants felt the need to take action following these results and what motivated or limited their actions (question 2). Finally, we describe the actions they considered (question 3). The results are enriched with participant’s quotes to provide more context.

### Views on the contents of the ISCOPE screening questionnaire

In general, participants agreed that the questionnaire’s four health domains (somatic, functional, social and psychological) covered the most important aspects of health for older persons and that health domains are interconnected as is stated by this 83-year-old male: “A*ll four (ed. the domains) are important. … And I can’t say, ‘No, I think one is more important than the other’ … They are all connected. That is it indeed, it’s your entire lifestyle.”*

Some participants felt that the social or psychological domains were most important. Also mentioned was difficulty with answering certain questions due to the limited answering options and limited space to add comments, as is explained by this 87-year-old female: “*Because you run into questions that you could really answer in two or more ways. And I find it difficult that you can only answer with crosses, because sometimes you’ve got a comment you want to add and there is never any space for it.”*

According to one participant, she actually felt worse than could be indicated on the questionnaire since there was no possibility to further elaborate on her answers.

The results were also influenced by experiences on a given day and could change per day or week. This is illustrated by this 87-year-old female: “*Well, yes the … about your health, sometimes you have pills, sometimes something needs to be done in the meantime, and then, then everything is fine again. But, yes, you give a depiction of that exact moment.”*

### Feedback on ISCOPE questionnaire results

Participants were interested in their current and previous results, and although not surprised by outcomes, these outcomes were seen as a useful confirmation of their own ideas regarding their personal health status, as is shown by the reaction of this 83-year-old female: “*Yes, I’m actually very happy with it, because now I’m discovering that the feeling that I am much better and can function a lot better is actually true.”*

Participants also felt that the results confirmed the positive effect of actions taken, or acted as a useful comparison with other older persons. They also appreciated that it allowed follow-up on long-term changes, as was mentioned by one 87-year-old female: “*And I also think it’s very good, if that is indeed the goal, that there’ll be another questionnaire later on, because then there is a lot in the old one … some things will have changed in your aging body …*. U*h … was it 2 or 3 years ago that I participated? And now it is today, for example, and there have definitely been some changes.”*

Participants appreciated feedback concerning their personal health, especially as they had invested time in completing the questionnaires. Nevertheless, two participants were not interested in their personal results, stating that they only participated for the benefit of other older persons.

Participants stated that they could imagine that the results of the questionnaire would be more relevant to them if at some point in the future they no longer had a good general idea of their own health situation. Others suggested that the results might be of value to other older persons who might lack an overview, as suggested by this 87-year-old female: “*I think that if it appears that someone can’t arrange everything for themselves anymore … , then it is important that the GP takes the initiative to bring about positive change to help that person regain the initiative to think about themselves and find a social network where they feel happy.”*

It was also suggested that the questionnaire could serve as an starting point for a General Practitioner (GP) or practice nurse to begin a conversation with a patient regarding their health and well-being in general, rather than focusing on specific complaints. Two participants made the following comments:

87-year-old male: “*Of course. Yes, well look, when you go to the GP those are all just single instants, right. Your illness or your pain or whatever. And he gives his answer and that is it, but really telling your story or really talking to someone? Because behind you is another patient. [I see. And how could a questionnaire help?] Well, of course, I gave a certain opinion or a certain answer. Based on those answers and the fact that the GP has known you for years there are little things that come up, like the chat like we’re having now, and then he will say, ‘really, I never expected that’.”*

88-year-old female: “*I think it is nice that the GP receives this information [ed. The results of the questionnaire]. You know, you only have ten minutes when you see the GP. Well, some things just don’t come up in conversation and that is understandable. You go there because you’ve got a problem and now he will have a better idea about you, about things that otherwise wouldn’t have come up.”*

Participants who had a good relationship with their GP doubted whether the questionnaire would reveal new information to their GP, as is explained by this 83-year-old male: *Oh well, I don’t know whether he [ed. the GP] can do anything with that [ed. the results from the questionnaire], because he, of course, already knows about everything as we are in regular contact.*

### Reasons (not) to take action

In general, participants were not inclined to undertake additional steps based on the results of the ISCOPE screening questionnaire, which could be explained by the fact that they were already aware of problems. They no longer reported problems they had previously acted on or they were either already acting on points raised by the questionnaire (e.g. physical therapy or a walking aid), as this 87-year-old female had done during recovery from surgery: “*I have also got a new knee. Yes, that means recovery but doesn’t mean you can’t do anything. I was still able to do everything regardless [laughs]. I just had to walk with a cane or crutches for a while, but the rest was all doable. … Yes. I am solution-oriented, yes.”*

A lack of motivation to improve general or specific health issues (i.e. physical health, memory issues) was also mentioned, as some participants felt that change was no longer possible at ‘their age’. These two participants explain:

Female, 87 years old: “*I don’t know whether this will ever be over; that I can’t do what I want …*. I *hope that this will all lessen a bit eventually, but I don’t really believe it …*”.

Female, 88 years old: “*No, it’s just that … well, you’re limited in everything. You can’t do as much anymore. I used to pour coffee sometimes, on one of those coffee mornings. Well, I don’t do that anymore, I can’t do that anymore. … It’s just because of old age”.*

Participants also stated that this situation might have been different a few years earlier.

### Possible measures to improve health situation

If participants were willing to invest in improving their personal health situation (based on current or worse than expected hypothetical results), they found it difficult to formulate possible measures themselves and preferred to receive suggestions in person from a general practice professional. It was also suggested that this could be integrated with the meeting in which screening questionnaire results were explained. Alternatively, participants could discuss serious results with their GP, providing they had a good relationship.

In contrast, this 87-year-old female only visits her GP when really necessary: “*Well, if it’s really necessary I visit her [ed. the GP] of course. But she’s often not there and that is quite a problem at the moment.”*

Participants also noted that it was unlikely that their GP would have time to discuss the results of the questionnaire, as this 87-year-old female said: “*It’s not terrible but to be honest I don’t really think that it [red. sending information to the GP] is necessary. [And could you indicate why you don’t feel it’s necessary?] Oh, that is very difficult to say. Yes … because the GP usually has far too little time whenever you visit the practice.”*

One participant (female, 87 years old) specifically mentioned that she wanted to decide for herself whether she discussed the results of the questionnaire with her GP: “*I have to do that myself, no, I don’t need help with that, I’ll do that myself. [Exactly, so it’s not that a questionnaire is sent to the GP and that …*] *No, the GP doesn’t need to get the results, I should do that myself. Yes, definitely, I’ll do that myself.”*

In general, participants agreed that it might be beneficial to discuss indications of serious health problems with their family, especially if these problems were unexpected and/or had changed considerably since the previous questionnaire. However, it was also mentioned that they did not plan to discuss their complaints with their children because they did not wish to bother them. A written overview of the results might also be helpful to the participant or their family. One participant (female, 86 years old) suggested that discussing the results with small groups of older persons would allow comparison and the sharing of ideas: “*… the group [ed. results] as well, because then you can start thinking that person has this and that person has that, and he is thinking about this and he has got that idea or plan. … I don’t know whether that would be possible, that the group could meet up? And that we could talk to each other about it, with guidance from the researchers.”*

## Discussion

Greater involvement of older persons in their personal health could increase self-management and help them benefit fully from proactive care. In this study we examined the attitudes of older persons to the outcomes of their screening questionnaires and their potential subsequent actions to improve or stabilize their health. Older persons acknowledged that a self-report screening questionnaire such as the ISCOPE screening questionnaire can potentially capture their current health situation. They were interested in direct feedback regarding their personal results on this screening questionnaire and in subsequent advice on possible additional measures. They were often disinclined to take additional actions themselves as a direct consequence of screening questionnaire results, since they often felt that they had already taken precautions. General practices could consider inviting older persons to complete a screening questionnaire and discuss personal goals and activities to attain these goals. Another suggestion was that small groups of older persons might discuss common experiences and possible measures.

### Comparison with literature

Several large trials on proactive care amongst community-dwelling older persons have not shown the expected benefits [11, 13] and the interventions did not lead to improvement of functioning in daily life. These interventions might have been more effective if older persons had been more involved in goal setting and decision making concerning their own health. Patient involvement and goal setting is gaining in importance in healthcare, especially for patients with chronic conditions that impact day-to-day life [10, 14]. These factors may well be key to the implementation of effective proactive care for older persons. Research has shown that patient involvement is difficult to implement in general practice [15, 16], that patient preferences vary widely [4, 17, 18] and that older patients generally tend to be less involved compared to younger patients [4, 5]. In addition to the availability and willingness of general practice professionals to invest time in patient involvement and shared decision making, the capacity and willingness of patients also demands attention [19]. In this study we examined older persons’ attitudes towards the results of their screening questionnaires and their subsequent actions. In line with earlier studies, we found that they appreciate being informed about their own health situation, but that they are unlikely to take action themselves [17, 18]. They have generally either already taken action or they would welcome advice from a professional from their general practice. As health problems become more complex, more healthcare professionals become involved and the question might arise as to which professional is the most suitable principal physician to initiate a conversation on health and wellbeing. In the Netherlands, the GP is the coordinating healthcare professional for most patients and it is therefore logical for the GP to take on this role. In most general practices in the Netherlands a practice nurse works with patients with chronic conditions and/or with older persons. An earlier study showed that older persons value the practice nurse [20], and a good relationship with their healthcare professional has been shown to encourage the open discussion of health problems [21, 22]. This opens up possibilities for reflection and discussion on health and wellbeing with older persons, with an important role for the practice nurse.

Those participants who had already taken action often suggested that they would most likely need additional guidance from a general practice professional as they grew older. This expectation is supported by research showing that patient involvement changes with health status [4, 18, 20]. Professionals at general practices should be aware of these differences; some older patients are fully capable of organizing healthcare themselves, while others may rely on the initiative of their GP or practice nurse. This poses extra challenges for GP practices [22, 23]. It would be interesting to further explore these differences in a new study on this topic. In addition, differences between individuals with regard to their socioeconomic status, the level of education, (chronic) disorders and treatment might be relevant to their opinion on this topic and an interesting subject for further study. Involving patients in decision making is a challenge that requires continuous effort from healthcare professionals in order to "help patients gain knowledge, skills, tools and confidence to become active participants in their care [24]" as stated by Bennett et al [24, 25]. This entails a different set of attitude and skills (i.e. "identifying values and goals of care" [25]) than that of the medical expert [22, 25]. It also requires that patients are aware of the possibility of discussing not only their medical health status, but also their personal goals with their GP or practice nurse [22, 26]. It will be interesting to explore methods to identify these personal goals and the skills general practice professionals need to help patients set and attain goals. When integrating shared decision making in general practice, the dynamic shared decision model suggested by van der Pol et al. might be an option. This model suggests a continuous dialogue between patient and healthcare professional in which goals, choices and treatment aims are discussed before decisions are taken and a treatment plan composed. If necessary, steps can be revisited [25].

### Strengths and limitations

This qualitative study entailed interviewing 13 community-dwelling older persons, all aged 80 years or older. Both those with and without complex problems according to the ISCOPE screening questionnaire were invited to participate, and all of those involved were able to participate in a conversation about their own health situation. As our focus was enhancing self-management, persons with cognitive problems were not included. In order to facilitate an open conversation about health, either one or two researchers spoke to all participants in their own homes.

As these interviews were not performed as part of usual care by the general practice, we did not have access to the participants’ medical files, we did not discuss their situation with their GP or practice nurse and we did not initiate any interventions. One possible advantage of this approach is independence, allowing participants to share any thoughts and ideas with us. We also noticed that participants had difficulty with some of the hypothetical questions in the interview. However, this aspect also stimulated participants to generate ideas regarding the potential benefits of the screening questionnaire for older persons lacking an overview of their personal health.

### Practice implications

This study showed that older persons value a chance to reflect on their health and functioning by means of a questionnaire, and to discuss possible measures with a professional at their general practice. A screening questionnaire could be integrated into general practice as a tool to support the discussion of personal goals and activities. Some older persons may not be interested since they are already aware of their (health) problems and have taken action or are already in regular contact with their GP or practice nurse. Others, however, might benefit from discussing their results and subsequent ideas on how to improve their health. GPs or practice nurses could support and motivate patients to think about their own personal goals [27, 28], but should also discuss whether and to what extent an older person wishes to be involved in his or her own health management [17, 20].

## Conclusions

Older persons were mainly interested in their own results as a confirmation, via an objective questionnaire, of their personal perceptions of their health. However, the results did not encourage participants to undertake additional actions since they felt that the questionnaire revealed no new information. Moreover, many experienced difficulty formulating additional measures to further improve or stabilize their current health situation. One possible action could be to discuss potential problems with their family or with a general practice professional. This knowledge can be used by general practices to increase the involvement and self-management of older persons by inviting them to discuss their health and well-being. A screening questionnaire could serve as an important tool to start this conversation. Older persons who struggle to maintain an overview of their personal health might derive the greatest benefit from discussing the results and potential subsequent actions to improve or stabilize their health situation. Further tailoring this method to involve older persons in their own health is a relevant topic for new research.

## Supplementary information

**Additional file 1.** Appendix 1. ISCOPE screening questionnaire.

**Additional file 2.** Appendix 2. Interview guide (translated from Dutch).

## Data Availability

The datasets used during the current study are, with restrictions due to privacy issues, available from the corresponding author on reasonable request.

## Abbreviations

ISCOPE: Integrated Systematic Care for Older PEople
GP: General practitioner
