# Supplementary material for: Older individuals’ views on their personal screening results for complex health problems: a qualitative study
Source: BMC Fam Pract. 2020 Oct 19;21:213. doi: 10.1186/s12875-020-01280-0 (PMC7574169; doi:10.1186/s12875-020-01280-0)
Supplement: Supplementary file 1 — Additional file 1. Appendix 1. ISCOPE screening questionnaire. [file 12875_2020_1280_MOESM1_ESM.docx]

**Appendix 1. ISCOPE screening questionnaire**

**Daily life abilities**

These first questions relate to how you function/manage your day-to-day life.

You may be helped in these activities by aids such as a stick, walking frame, or wheelchair.

1. Can you do the shopping without help from anyone else?

*Yes / No*

2. Can you walk outdoors without help from anyone else?

*Yes / No*

3. Can you dress and undress yourself without help from anyone else?

*Yes / No*

4. Can you go to the toilet without help from anyone else?

*Yes / No*

5. Can you manage your finances yourself (collect your money, pay your bills)?

*Yes / No*

6. How well would you say you cope with your general day-to-day life?

*Well / Average /Not at all well*

**Health and illness**

7. Which mark would you give for your physical fitness?

*1 2 3 4 5 6 7 8 9 10*

*Not at all fit Very fit*

8. Do you experience day-to-day problems due to poor eyesight (even if you wear glasses or contact lenses)?

*Yes / No*

9. Do you experience day-to-day problems due to poor hearing (even if you wear a hearing aid)?

*Yes / No*

10. Do you experience problems with incontinence of urine or stool?
*Yes / No*

11. Do you experience daily problems due to pain?
*Yes / No*

12. Have you lost weight (more than 6 kg) in the last 6 months unintentionally?

*Yes / No*

13. Are you using more than 4 different kinds of medicine at the moment?

*Yes / No*

14. Have you had a fall in the last month?

*Yes / No*

15. Have you been admitted to the hospital in the last 6 months?

*Yes / No*

**Psychological functioning**

16. Do you feel you have memory complaints?

*Yes /Sometimes/ No*

17. Have you recently felt sad or depressed?

*Yes /Sometimes/ No*

18. Have you recently felt nervous or anxious?

*Yes /Sometimes/ No*

19. Do you feel pretty worthless at the moment?

*Yes /Sometimes/ No*

**Social functioning**

20. Do you feel that your life is empty?

*Yes /Sometimes/ No*

21. Do you feel the lack of a close friend?

*Yes /Sometimes/ No*

22. Do you feel left alone sometimes?

*Yes /Sometimes/ No*

23. Do you feel there are enough people with whom you feel a close connection?

*Yes /Sometimes/ No*

24. Do you receive help from anybody in you immediate surrounding because you are unable to do things for yourself?
*Yes / No*

25. Has anyone helped you to fill in this questionnaire?
*No, I have filled in the questionnaire myself.
Yes, someone helped me to answer these questions.
Somebody has answered the questions for me.*

26. At the moment, which health complaints limit you the most in your day-to-day life?
