# Supplementary material for: Older individuals’ views on their personal screening results for complex health problems: a qualitative study
Source: BMC Fam Pract. 2020 Oct 19;21:213. doi: 10.1186/s12875-020-01280-0 (PMC7574169; doi:10.1186/s12875-020-01280-0)
Supplement: Supplementary file 2 — Additional file 2. Appendix 2. Interview guide (translated from Dutch). [file 12875_2020_1280_MOESM2_ESM.docx]

**Appendix 2. Interview guide (translated from Dutch)**

Introduction of the interviewer and explanation of the study. Important: interviews are based on the participant’s personal results on the ISCOPE screening questionnaire (for research purposes), an audio recording will be made, and anonymous data published in a research article.

**Turn on recording:** state name and date and ask again for consent (as explained earlier)

1. **Experience of the ISCOPE screening questionnaire**

Complete picture of your health?

Relevant questions?

A questionnaire to form a picture of your health: a good idea?

Results should be available to participant? GP? Both? Others?

What if results are very good/bad?

**Provide results of the current questionnaire and compare to previous questionnaires**

1. **Receiving the results**

Understanding the results?

Importance of parts of the questionnaire?

Value of receiving this information?

1. **Perception of results**

Per health domain: surprise? Recognition?

Reason for differences with last year?|

Complete picture of your health?

Relevant questions?

1. **Taking action**

Per health domain: inclined to take action?

Previous actions taken? Support from network?

Ideas on prevention of functional decline or improvement of health situation?

Talking about the results with…?

Close-ended question: take action themselves/to GP/help from family

Results useful?

1. **Necessity to take action** [take action/feel better/improve results/improve health]

Why take (no) action?

What is required to be able to or want to take action?

What/who can help you actually take action?

Preference regarding how results are received?

1. **Relationship with GP** [can be discussed earlier if appropriate]

Experiences of sharing problems

Confidence in their GP

Results are a reason (not) to contact their GP?

1. **Considerations if results were different**

What actions would be considered in case of a much worse/better result?

Thank you for everything you’ve shared. Anything to add? **Stop recording**
